# Solitary rib recurrence of hilar cholangiocarcinoma 10 years after resection: report of a case

**DOI:** 10.1007/s12328-013-0432-8

**Published:** 2013-11-08

**Authors:** Yohei Ota, Ryusei Matsuyama, Koichi Taniguchi, Michio Ueda, Kazuhisa Takeda, Kuniya Tanaka, Takashi Nakayama, Itaru Endo

**Affiliations:** 1 Department of Gastroenterological Surgery, Yokohama City University Graduate School of Medicine, 3-9 Fukuura, Kanazawa-ku, Yokohama, 236-0004 Japan; 2 Pathological Department, Yokohama City University Graduate School of Medicine, Yokohama, Japan

**Keywords:** Cholangiocarcinoma, Long-term survivor, Rib recurrence, Positron emission tomography, Carbohydrate antigen 19-9

## Abstract

A 61-year-old female underwent right hemihepatectomy and caudate lobectomy for hilar cholangiocarcinoma in 1999. Ten years later, increasing serum carbohydrate 19-9 was detected by routine follow-up. Subsequent positron emission tomography revealed an asymptomatic lesion in the right 11th rib. As the mass steadily grew in size, the lesion was resected en bloc with the affected rib and muscle. The histopathological findings closely resembled those of the primary cholangiocarcinoma. Thus, the tumor was diagnosed as a metastatic recurrence 10 years after resection of the primary tumor. There have been a few reports of cholangiocarcinoma recurrence in long-term survivors at the surgical margins, peritoneum, or transhepatic drainage route. However, there are no reports of solitary extra-abdominal recurrence. This case highlights the need for careful follow-up of patients with cholangiocarcinoma and nodal metastasis, even in the absence of recurrence for >5 years after curative resection.

## Introduction

Bile duct cancer has been associated with a poor prognosis; node metastasis is a particularly important poor prognostic factor [1–6]. Despite advances in the diagnosis and treatment of cholangiocarcinoma, such as endoscopic biliary drainage, surgical procedures, and chemotherapy, long-term outcomes remain unsatisfactory because of high rates of disease recurrence. Recurrence of bile duct cancer usually occurs within 5 years after surgery. Several reports of recurrence in long-term survivors have also been published [7–9]. Recurrence usually occurs at the margin of the bile duct, in the peritoneum, or in the liver [1, 2]. Solitary recurrences outside the peritoneal cavity are rare [10]. This report describes a patient with hilar cholangiocarcinoma who underwent resection of 11th rib metastasis 10 years after curative resection of the primary tumor.

## Case report

A 61-year-old female was diagnosed with hilar cholangiocarcinoma in 1999. It mainly affected the right hepatic duct, but also extended to the left hepatic duct and proximal common bile duct. Percutaneous transhepatic cholangio drainage tubes were inserted into the anterior brunch and posterior brunch approached from the 7th intercostal space and into the posterior brunch approached from the 9th intercostal space. The patient underwent right hemihepatectomy and caudate lobectomy after portal vein embolization. The tumor was 2.2 cm in diameter and infiltrated to the liver parenchyma. The cystic node and retroportal nodes were histologically involved. According to the sixth edition of the UICC TNM classification [11], the tumor was classified as pT3 pN1 pM0 pStage III. In addition, according to the fifth edition of the General Rules for Biliary Tract Cancer by the Japanese Society of Biliary Surgery [12], the tumor was described as Bcrism, nodular-infiltrating type, 2.2 cm, moderate- to well-differentiated adenocarcinoma (tub2 > tub1), pat, sci, infB, ly1, v0, pn0, hinf1, ginf0, panc0, du0, hm1, dm1, em1, pv0, a0, n2 (12c 1/1, 12p 1/8), pT4 pN2 M0 fStage IVb, final curability B. Four months after the initial operation, the patient underwent eight courses of hepatic arterial injection of fluorouracil (5FU) (1,500 mg) every week as adjuvant chemotherapy. Thereafter, three courses of systemic adjuvant chemotherapy with methotrexate (50 mg), cisplatin (20 mg), and 5FU (750 mg) on days 1, 8, and 15 were administered every 6 months, along with radiation therapy to the hilar region at a dose of 56 Gy. Written informed consent was obtained prior to chemotherapy. After these adjuvant therapies, the patient continued to be followed up with serum carcinoembryonic antigen (CEA) and carbohydrate antigen 19-9 (CA 19-9) testing every 2 months and computed tomography (CT) every 6 months for 10 years; she showed no signs or symptoms of recurrence over the next 10 years.

**Fig. 1:**
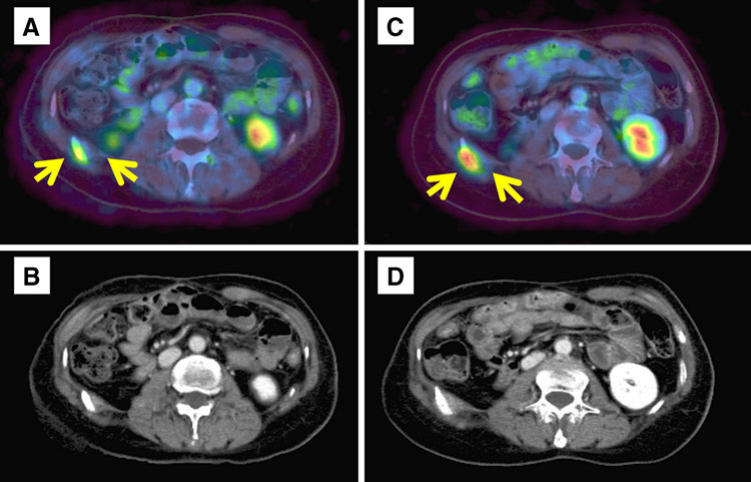
Progression of the tumor on PET and CT. PET and CT show a gradual increase in tumor size. **a**, **b** September 2008. **c**, **d** April 2009

In September 2008, a sudden increase of the serum CA19-9 level to 68 U/mL (normal range <37 U/mL) was noted. The site of recurrence could not be identified on CT. Positron emission tomography (PET) revealed an elevated standard uptake value of 3.4 in the 11th right rib. We chose the wait-and-watch approach because of the low suspicion of metastasis and the patient’s wishes. The wait-and-watch approach was adopted in view of the absence of any bone destruction, mass formation, or symptoms. In February 2009, the serum CA19-9 level increased from 68 to 207 U/mL. PET again revealed a solitary accumulation in the 11th right rib and adjacent tissues, with an increase of the standard uptake value from 3.4 to 5.8 (Fig. 1). There were no other lesions detected by PET or any other examinations, including episode of rib fracture, upper gastrointestinal endoscopy, colonoscopy, and mammography. These findings were suggestive of bone metastasis 10 years after curative resection of the primary cholangiocarcinoma.

In April 2009, a tumor biopsy was performed under general anesthesia by an orthopedic surgeon. Intraoperative frozen section diagnosis revealed adenocarcinoma, and tumor resection was carried out en bloc, along with the 11th rib, adjacent intercostal muscle, and surrounding tissues.

**Fig. 2:**
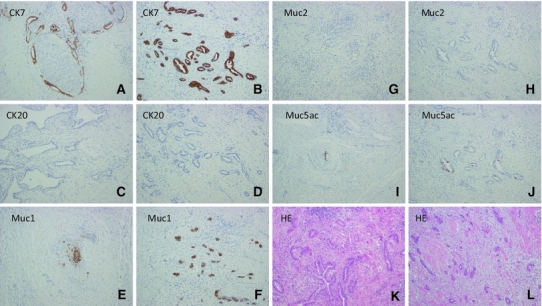
Immunohistochemical examination showed the staining pattern of the primary bile duct cancer to be the same as that of the thoracic tumor. Both the primary cancer (**a**, **c**, **e**, **g**, **i**, **k**) and thoracic tumor (**b**, **d**, **f**, **h**, **j**, **l**) were well stained by CK7 (**a**, **b**), CK20 (**c**, **d**), MUC1 (**e**, **f**), MUC2 (**g**, **h**), MUC5ac (**i**, **j**), and H&E (×100)

Histopathological examination of the resected specimen revealed metastatic adenocarcinoma of the costal bone with invasion of the adjacent intercostal muscle. The tumor morphology closely resembled that of the resected primary bile duct cancer. An immunohistochemical study was performed (Fig. 2), which revealed an identical staining pattern in both tumors. Both tumors showed positive staining for CK7, MUC1, and MUC5ac and negative staining for CK20 and MUC2. Based on these findings, it was concluded that the 11th right rib tumor was a recurrence of the hilar cholangiocarcinoma.

Over a period of eighteen months after the second surgery, while the patient was observed without systemic chemotherapy, the serum CA19-9 level decreased. CT and PET showed no evidence of recurrence; therefore, close follow-up has continued.

## Discussion

Long-term results of patients with cholangiocarcinoma, especially with nodal metastases, are unsatisfactory. Several investigators have reported a mean survival time of 12–22 months in patients with nodal metastasis [1, 4, 6]. Disease recurrence in the liver and peritoneum usually occurs within 3 years after the operation [13–15]. Several reports of recurrences in long-term (>5 years) survivors after curative resection for intrahepatic bile duct cancer have been published [7–9]. In most of these cases, the recurrence occurred at the surgical margins of the bile ducts or in the peritoneum(Table 1). These reports were subject to intrahepatic bile duct cancer, but no report has published in hilar or extra bile duct cancer. Solitary recurrences outside the peritoneal cavity are not common, and bone metastasis is rare [10]. In terms of other gastroenterological cancers, long-term survival rates have been reported in only a few studies [16–18]. For gastric cancer, Moon et al. [18] reported that a ≥10-year long-term recurrence after surgery occurred in 2.0 % of patients. In terms of the recurrence pattern, peritoneal carcinomatosis was dominant within 5 years post-gastrectomy. In contrast, distant metastasis was the main relapse pattern during 5–10 years post-gastrectomy and after 10 years post-gastrectomy. Until now, there have been no reports of bone metastasis in long-term survivors by PubMed searches using ‘cholangiocarcinoma’, ‘bone metastasis’, and ‘long-term survivor’ as searchable terms in any entire period.

**Table 1:** The recurrence of bile duct cancer was detected in long-term survivors >5 years after the curative resection

No.	Author	Age (years)/gender	Length (years)	Primary lesion	Pathology	Adjuvant chemotherapy	Location of recurrence
1	Machimotom	74/F	12	Hilar	tub1	Oral UFT for 5 years	Abdominal wall
2	Sasaki	45/M	9	MBD	tub2	No	Choledochojejunostomy region
3	Tanaka	53/M	10	LBD	pap	No	Choledochojejunostomy region
4	Our case	60/F	10	Hilar	tub2	HAI UFT 4 weeks for 1 year	11th rib
						MTX + CDDP + 5FU 6 months for 1 year 6 months with radiation for 56 Gy	

*MBD* middle bile duct, *LBD* lower bile duct, *UFT* uracil–tegafur, *HAI* hepatic arterial injection, *MTX* methotrexate, *CDDP* cisplatin, *5FU* fluorouracil

Recurrences many years after the treatment may be related to long-lasting tumor dormancy being turned on, especially in distant organs [19–21]. Holmgren et al. [19] reported that the tumor dormancy theory is defined as ‘no change’; the tumor does not disappear, but remains at the same size for a long period of time. In many types of cancer, improvements in outcome have been reported when the tumor is in an immutable state. Takahashi et al. [20] reported that tumor dormancy is induced by chemotherapy, and patients can survive for long periods without signs of disease recurrence, even if the tumor remains. In our case, it might be estimated that adjuvant chemotherapy induced tumor dormancy; therefore, our patient was alive without recurrence for a long period of time. Surgical stress might also be associated with tumor metastasis [22]. In this case, although solitary rib metastasis was apparent, some other dormant cancer cells might exist systemically and be activated by the surgical stress. Kato et al. [23] reported efficacy of downsizing chemotherapy in unresectable locally advanced cholangiocarcinoma. These findings seemed to have a choice of resection if the tumor was controlled by chemotherapy several times. Our case was not strongly suspicious of metastasis, but chemotherapy may have been a reasonable strategy if cancer had been confirmed in biopsy.

Otherwise, the immune system is able to effectively mobilize against tumor invasion. Steffen et al. [24] reported that in melanoma, the tumor–immune system dynamic is critically important in determining tumor regrowth after resection. Adjuvant immunotherapy using polysaccharide-K (Krestin) reportedly had a survival benefit in gastric and colorectal cancer, and the host immune status was considered to be one of the prognostic factors [25]. More recently, in ovarian cancer, neurobehavioral stress [26] and stress-associated hormones such as norepinephrine, epinephrine, and cortisol [27] have been shown to be associated with increased tumor growth and metastasis. Tumors may rapidly grow from various causes, such as changes in immune function and physical condition. In this case, however, there was no deterioration of immune function, weight loss, or deterioration of nutritional status in the 10 years that the patient was observed. Such cases keenly highlight the difficulty of achieving cures in patients with bile duct cancer. Although several possibilities have been considered, factors predictive of long-term recurrence of bile duct cancer remain unclear.

In cholangiocarcinoma, CA19-9 is elevated in 50–79 % of patients [28]. The diagnostic potential for primary lesions is almost equivalent between PET and CT, whereas PET is superior in the diagnosis of new metastatic lesions [29]. Anderson et Al. reported that 30 % of patients evaluated for suspected cholangiocarcinoma had their therapy plans altered because of detection of unsuspected metastases on FDG-PET [30]. The present patient was followed up for >10 years after curative resection of the primary tumor because she had positive nodal metastasis. Close follow-up was performed for >5 years after the initial operation with serum CA19-9 testing every 2 months and CT every 6 months. In this case, the metastatic lesion was suspected based on the elevation of CA19-9. Thus, solitary rib metastasis, which could not be found by CT, was detected by PET. Long-term survival patients with cholangiocarcinoma may require continual long-term surveillance of tumor markers such as CEA or CA19-9, and active implementation of PET might be beneficial when these tumor markers are elevated.

In conclusion, in patients with bile duct cancer, it must be emphasized that long-term surveillance is required even in patients without recurrence for >5 years after curative resection of the primary tumor, even if there are no early signs of recurrence.
